# Nationwide profiling of insecticide resistance in *Aedes albopictus* (Diptera: Culicidae) in Cameroon

**DOI:** 10.1371/journal.pone.0234572

**Published:** 2020-06-18

**Authors:** Aurelie P. Yougang, Basile Kamgang, Armel N. Tedjou, Theodel A. Wilson-Bahun, Flobert Njiokou, Charles S. Wondji

**Affiliations:** 1 Centre for Research in Infectious Diseases, Yaoundé, Cameroon; 2 Department of Animal Biology and Physiology, Parasitology and Ecology Laboratory, Faculty of Science, University of Yaoundé 1, Yaoundé, Cameroon; 3 Laboratory of vertebrate and invertebrate bioecology, Faculty of Science and Technology, Marien-Ngouabi University, Brazzaville, Congo; 4 Liverpool School of Tropical Medicine, Liverpool, United Kingdom; Faculty of Science, Ain Shams University (ASU), EGYPT

## Abstract

The Asian mosquito, *Aedes albopictus* (Skuse), is an invasive mosquito which has become one of the most important vectors of dengue, Zika, and chikungunya viruses worldwide. This species was reported for the first time in Cameroon in early 2000s and became the dominant *Aedes* species in the urban areas in the southern part of Cameroon but remain poorly characterized. Here, we assessed the susceptibility profile of *A*. *albopictus* collected throughout Cameroon and investigated the potential resistance mechanisms involved. Immature stages of *A*. *albopictus* were collected between March and July 2017 in 15 locations across Cameroon and reared until G1/G2 generation. Larval, adult bioassays, and synergists [piperonyl butoxide (PBO) and diethyl maleate (DEM)] assays were carried out according to WHO recommendations. F1534C mutation was genotyped in field collected adults (Go) using allele specific PCR. All tested populations were susceptible to both larvicides, temephos and *Bacillus thuringiensis israelensis* (*Bti*), after larval bioassays. Adult bioassays revealed a high level of resistance of *A*. *albopictus* to 4% DDT with mortality rates ranging from 12.42% in Bafang to 75.04% in Kumba. The resistance was reported also in 0.05% deltamethrin, 0.25% permethrin, and 0.1% propoxur in some locations. A loss of susceptibility to 0.1% bendiocarb was found in one of three populations analysed. A full susceptibility to 1% fenitrothion were observed across the country. A full recovery or partial of susceptibility was observed in *A*. *albopictus* when pre-exposed to PBO or DEM and then to DDT and permethrin, respectively. The F1534C *kdr* mutation was not detected in *A*. *albopictus*. This study showed that the susceptibility profile of *A*. *albopictus* to insecticide vary according to the sampling location and insecticides used. These findings are useful to planning vector control program against arbovirus vectors in Cameroon and can be used as baseline data for further researches.

## Introduction

The Asian tiger mosquito, *Aedes albopictus* (Skuse) 1894, originated from South East Asia has invaded all the five continents during the past four decades [1]. This mosquito species is one of the most important vectors of several arboviruses including dengue virus (DENV, *Flaviviridae*, *Flavivirus*) [2], chikungunya virus (CHIKV, *Togaviridae*, *Alphavirus*) and Zika virus (ZIKV, *Flaviviridae*, *Flavivirus*) [3,4] worldwide. During the last two decades, diseases caused by these viruses are increasingly reported in several regions of the world including Central Africa [3,5–13] where the epidemics were formerly considered as scarce. Coincidentally, the emergence of arboviral diseases notably dengue and chikungunya has matched with the establishment of *A*. *albopictus* in the region. Indeed, *A*. *albopictus* was reported for the first time in Central Africa in Cameroon in early 2000s [14], and has rapidly colonized almost all countries of the subregion [15]. Nowadays, in Cameroon *A*. *albopictus* is the dominant *Aedes* species in most cities located under 6 N latitude [16]. This species was suspected as the main vector during dengue and chikungunya outbreaks in Cameroon in 2006 [6]. It was also recently demonstrated that *A*. *albopictus* populations collected in different ecological settings in Cameroon are able to transmit dengue 2 virus [17].

Control of *A*. *albopictus* relies on destruction of breeding sites and insecticide-based interventions. Indeed, the use of larvicides such as *Bacillus thuringiensis var*. *israelensis* (*Bti*) or temephos to treat water storage containers and space spraying of adulticides in emergency situations can help to reduce the density of *Aedes* mosquitoes [18,19]. Unfortunately, intensive and prolonged use of insecticides usually leads to the emergence of resistance in mosquito species under selection pressure, by decreasing the frequency of susceptible mosquitoes and reducing variability of field mosquitoes [20]. Thus, many vector control programmes are facing the challenge from the development of insecticide resistance in *A*. *albopictus*. Two main mechanisms are associated in insecticide resistance: target site due to the mutation that reduces or blocks the binding affinity between the insecticide and target site [21], and metabolic resistance [22]. Target site resistance is caused by the mutation in target genes such as the acetylcholinesterase (*Ace-1*), the *GABA* receptor, and the voltage-gated sodium channel (*VGSC*) causing knockdown resistance (*kdr*). Among target site resistance, *kdr* resistance is one of the main conferring resistance to both pyrethroids and dichlorodiphenyltrichloroethane (DDT). In *A*. *albopictus*, *kdr* mutation is less prevalent with only four *VGSC* mutations detected affecting two codons (1532 and 1534). Among these mutations only the F1534S variant has been shown to be moderately associated with resistance to DDT and pyrethroids [23,24]. Furthermore, metabolic resistance through upregulation of detoxification genes is a common resistance mechanism in *A*. *albopictus*. It is caused primarily by three main enzyme families, the monooxygenases (cytochrome P450s), glutathione S-transferases (*GSTs*) and carboxylesterases (*COEs*) [22,25]. *CYP6P12* gene has been shown to be over-expressed in pyrethroid resistant *A*. *albopictus* in Asia [26]. In Cameroon, data on insecticide resistance in *A*. *albopictus* are very scarce apart preliminary studies which highlighted the high resistance of this species to DDT, and a loss of susceptibility to pyrethroids and carbamates [27,28]. As insecticide resistance is a dynamic process which can vary according geographical space and time, we undertook this study aiming to assess the susceptibility profile of *A*. *albopictus* nationwide and the potential resistance mechanisms involved.

## Materials and methods

### Ethics statement

This study was approved by the Cameroonian national ethics committee for human health research N˚2017/05/911/CE/CNERSH/SP including using of rabbits. Oral consent to inspect the potential breeding sites was obtained in the field from household or business occupants.

#### Mosquito sampling

**Fig 1:**
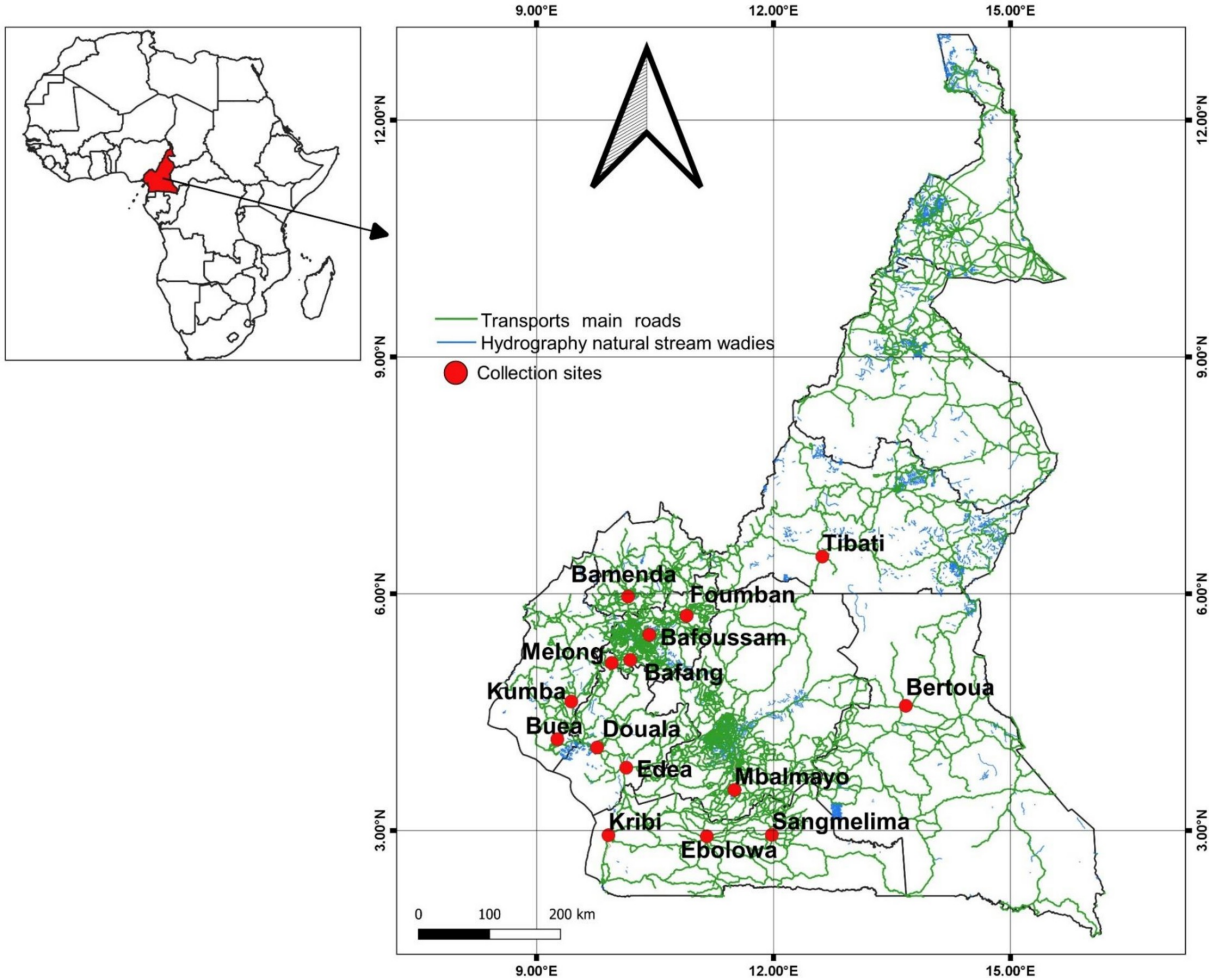
Map of Cameroon showing the sampling sites of *Aedes albopictus*.

*A*edes *albopictus* mosquitoes were sampled as larvae or pupae between March and July 2017 in 15 locations across Cameroon (Fig 1): Edéa (03°48’N; 10°08’E), Buea (04°09’N; 09°14’E°), Bafang (05°09’N; 10°14’E), Bafoussam (05°28’N; 10°25’E), Bamenda (05°56’N; 10°10’E), Sangmelima (02°56’N; 11°58’E), Ebolowa (02°54’N; 11°09’E), Mbalmayo (03°31’N; 11°30’E), Bertoua (04°33’N; 13°46’E), Kribi (02°57’N; 09°55’E), Kumba (04°38’N; 09°27’E), Tibati (06°28’N; 12°38’E), Foumban (05°43’N; 10°55’E), Melong (05°07’N; 09°57’E), and Douala (04°03’N; 09°42’E).

Immature stages (field generation, G0) were collected from different potential breeding sites: domestic (e.g. jars, tanks), peri-domestic (e.g. used tires, discarded tanks), and natural (e.g. tree holes). In each location, larvae or pupae from 20 positive larval breeding places were collected, stored in plastic boxes and transferred to insectary, pooled according to the location and reared to adult stage for identification using taxonomic keys [29,30]. Mosquitoes identified as *A*. *albopictus* were reared until generation G1 for adult bioassays and G2 for larval bioassays. Mosquito populations were maintained at insectary conditions (27°C ± 2°C; relative humidity 80% ±10%), and females were fed on rabbits to complete their gonotrophic cycle. The *A*. *albopictus* susceptible strain from the Malaysia Vector Control Research Unit (VCRU) lab strain coming from Malaysia was used as reference strain.

#### Larval bioassays

Larval bioassays were performed according to WHO guidelines [31] using G2 generation larvae. The susceptibility of larvae was evaluated against technical grade temephos (Sigma Aldrich-Pestanal®, Seelze, Germany) and a formulated *Bacillus thuringiensis israelensis* (*Bti*) product (Vectobac® 12AS, Illinois, USA). First, stock solutions and serial dilutions were prepared in 95% ethanol for temephos and distilled water for *Bti* and stored at 4°C. Six doses of concentrations ranging from 0.08 to 0.2 mg/l for *Bti*, and from 0.001 to 0.006 mg/l for temephos have been used. 80 to 100 larvae per concentration (with three to four replicates, depending on the sample and the number of larvae available) were tested. Late-third or early-fourth instars larvae of *A*. *albopictus* were placed in plastic cups with 99 ml of distilled mineral water, and 1 ml insecticide solution at the required concentration was added.

Control groups were run systematically with larvae exposed to 1 ml of ethanol for temephos or 1 ml of distilled water for *Bti*. No food was provided to larvae during the bioassays, which were run at 27 ± 2°C and 80 ±10% relative humidity. Mortality was determined after 24 hrs of exposure to the insecticide. Mortality rates were corrected with Abbott’s formula [32], when the mortality of controls was > 5%. All data were analysed with Win DL v. 2.0 software [33]. Lethal concentrations (LC50 and LC95) were calculated with their 95% confidence intervals (CIs). Resistance ratios (RR50 and RR95) were calculated by comparing the LC50 and LC95 for each species with those of susceptible strain, as RR50(95) = LC50(95) of studied population/LC50(95) susceptible strain and RR95 = LC95(95) of studied population / LC95(95) reference strain. The resistance levels were ranked into three categories: susceptibility (RR_50_< 5), medium or moderate resistance (5 ≤ RR_50_ ≤ 10), and high resistance (RR_50_ > 10) [31].

#### Adult bioassays

Bioassays were carried out according to WHO protocol [31] using 3–5 days-old G1 generation *A*. *albopictus* mosquitoes with 4 replicates of 20–25 mosquitoes per tube. Six insecticides were tested: 0.25% permethrin (type I pyrethroid), 0.05% deltamethrin (type II pyrethroid), 4% DDT (organochlorine), 0.1%propoxur (carbamate), 0.1% bendiocarb (carbamate), and 1% fenitrothion (organophosphate). Insecticide-impregnated papers were supplied by Liverpool School of Tropical Medicine. Mortality was recorded 24 hrs later and mosquitoes alive and dead after exposure 24h were stored in RNA later and silica gel, respectively. The resistance status was defined as follows: susceptible (mortality rate between 98–100%), probable resistance (mortality rate between 90–98%), and resistant (mortality rate inferior to 90%) [31]. For populations (Bafang, Bafoussam, Tibati, and Edéa) which revealed a high level of resistance to diagnostic dose of permethrin (0.25%) recommended by WHO, an additional test was performed with 2x (0.5%) and 3x (0.75%) of the discriminating dose of permethrin. Four replicates of 20–25 females per tube were exposed to 0.25, 0.5, and 0.75% permethrin for 1 h. The *A*. *albopictus* strain from the Malaysia Vector Control Research Unit (VCRU) was used as reference susceptible lab strain.

#### Synergist assays

In order to investigate the potential role of oxidases and glutathioneS-transferases (*GSTs*) in the metabolic resistance mechanism, synergist assay was performed, when number of mosquitoes permitted, using 4% piperonyl butoxide (PBO) and/or 8% diethyl maleate (DEM) respectively. 3-5-days-old adults were pre-exposed for one hour to PBO- or DEM-impregnated papers and after that immediately exposed to the selected insecticide. Mortality was scored 24 hrs later and compared to the results obtained with each insecticide without synergist according to the WHO standards [34]. The comparison of mortality rate after pre-exposure of mosquitoes to synergist and without pre-exposure to synergist was done using Chi-square test. The difference was statistically different when *P-value* was inferior to 0.05.

#### Investigating of F1534mutation using allele specific PCR

Genomic DNA of 30 specimens of *A*. *albopictus* per populations was extracted using Livak protocol [35]. This DNA was used to genotype the F1534 mutation using allele specific (AS) PCR as described previously [36,37]. Each PCR reaction was performed using a Gene Touch thermal cycler (Bulldog Bio, Portsmouth, USA) in a 15 μl volume containing: 1 μl of DNA sample, 0.4 units of Kapa Taq DNA polymerase, 0.12 μl of 25 mM dNTPs (0.2 mM), 0.75 μl of 25 mM MgCl_2_ (1.5 mM), 1.5 μl of 10× PCR buffer (1×), 0.51 μl of each primers (0.34 mM). The amplification consisted of 95°C for a 5 min heat activation step, followed by 35 cycles of 94°C for 30 s, 55°C for 30 s and 72°C for 45 s with a 10 min final extension step at 72°C.PCR products were detected by agarose gel electrophoresis in Tris-Acid-EDTA buffer (TAE). The 3% gel was prepared with Midori green, staining dye, and visualized with the aid of UV light.

## Results

### Larval bioassays to *A*. *albopictus*

Due to the limited number of larvae available, larval bioassays were performed for six populations from Bertoua, Douala, Bafoussam, Tibati, Bafang, and Edéa with temephos, and four populations from Bertoua, Bafoussam, Tibati, and Edéa with *Bti* (Tables 1 and 2). Analysis revealed that for both larvicides and populations tested, RR_50_ and RR_95_ were less than 5 suggesting that *A*. *albopictus* from these locations are susceptible to *Bti* and temephos.

**Table 1:** Larval bioassays with temephos against *Aedes albopictus* larvae.

Strain and Site	N	LC_95_ (mg/L) (95% CI)	RR_95_	LC_50_ (mg/L) (95% CI)	RR_50_
VCRU	574	0.0068 (0.00512–0.0209)	-	0.0031 (0.00128–0.00413)	-
Bertoua	439	0.0073 (0.00695–0.00789)	1.07	0.0053 (0.00512–0.00547)	1.68
Douala	432	0.0120 (0.01007–0.0173)	1.75	0.0079 (0.00738–0.00921)	2.52
Bafoussam	508	0.0077 (0.0071–0.00856)	1.12	0.0043 (0.00421–0.00461)	1.38
Tibati	547	0.0055 (0.00440–0.00915)	0.80	0.0030 (0.00238–0.00360)	0.95
Bafang	523	0.0056 (0.00520–0.00641)	0.83	0.0031 (0.00291–0.00330)	0.98
Edéa	549	0.0066 (0.00589–0.00775)	0.97	0.0028 (0.00254–0.00320)	0.91

LC_95_ and LC_50:_ 95 and 50% lethal concentrations; CI: confidence interval; RR: resistance ratio; VCRU: Vector Control Research Unit.

**Table 2:** Larval bioassays with *Bti* against *Aedes albopictus* larvae.

Strain and Site	N	LC_95_ (mg/L) (95% CI)	RR_95_	LC_50_ (mg/L) (95% CI)	RR_50_
VCRU	574	0.0408 (0.0332–0.0546)	-	0.0108 (0.0096–0.0120)	-
Bertoua	548	0.0570 (0.0445–0.0893)	1.39	0.0169 (0.0139–0.0193)	1.56
Bafoussam	556	0.150 (0.0827–0.953)	3.67	0.0272 (0.0208–0.0342)	2.51
Tibati	547	0.0907 (0.0648–0.163)	2.22	0.0298 (0.0263–0.0359)	2.75
Edéa	526	0.0322 (0.0261–0.0426)	0.78	0.0088 (0.00302–0.0132)	0.81

LC_95_ and LC_50:_ 95 and 50% lethal concentrations; CI: confidence interval; RR: resistance ratio; VCRU: Vector Control Research Unit.

### Adult bioassays

Bioassays performed with susceptible strain revealed that this *A*. *albopictus* strain was fully susceptible to almost all insecticides tested excepted DDT for which 80.7% mortality rate were found.

**Fig 2:**
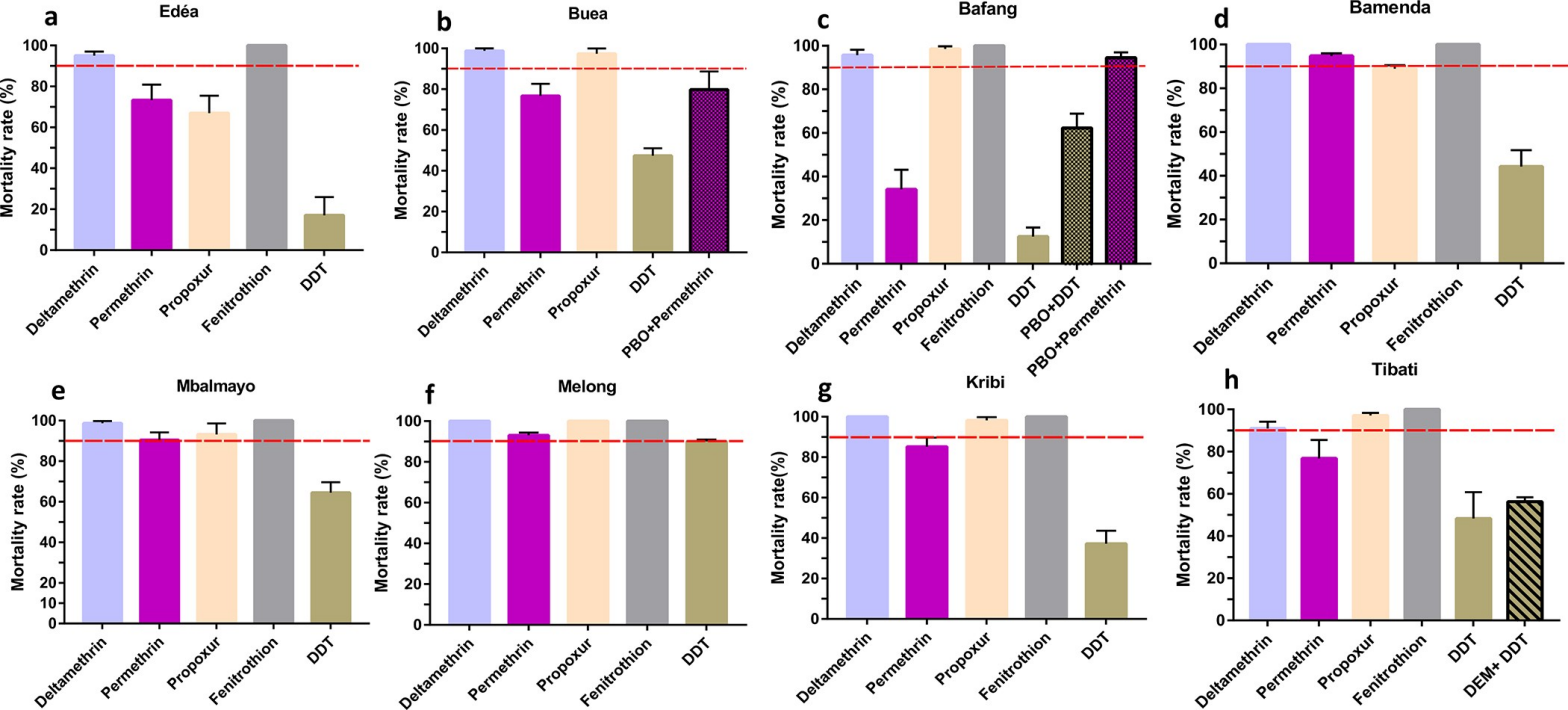
Mortality rates of adult *A*. *albopictus* from Cameroon (8 locations) 24 hrs after exposure to insecticides alone or with 1 h pre-exposure to synergist. a, Edéa; b, Buea; c, Bafang; d, Bamenda; e, Mbalmayo; f, Melong; g, Kribi; h, Tibati. Error bars represent standard error of the mean (SEM). DDT, Dichlorodiphenyltrichloroethane. PBO, Piperonyl butoxide. DEM, Diethyl maleate.

**Fig 3:**
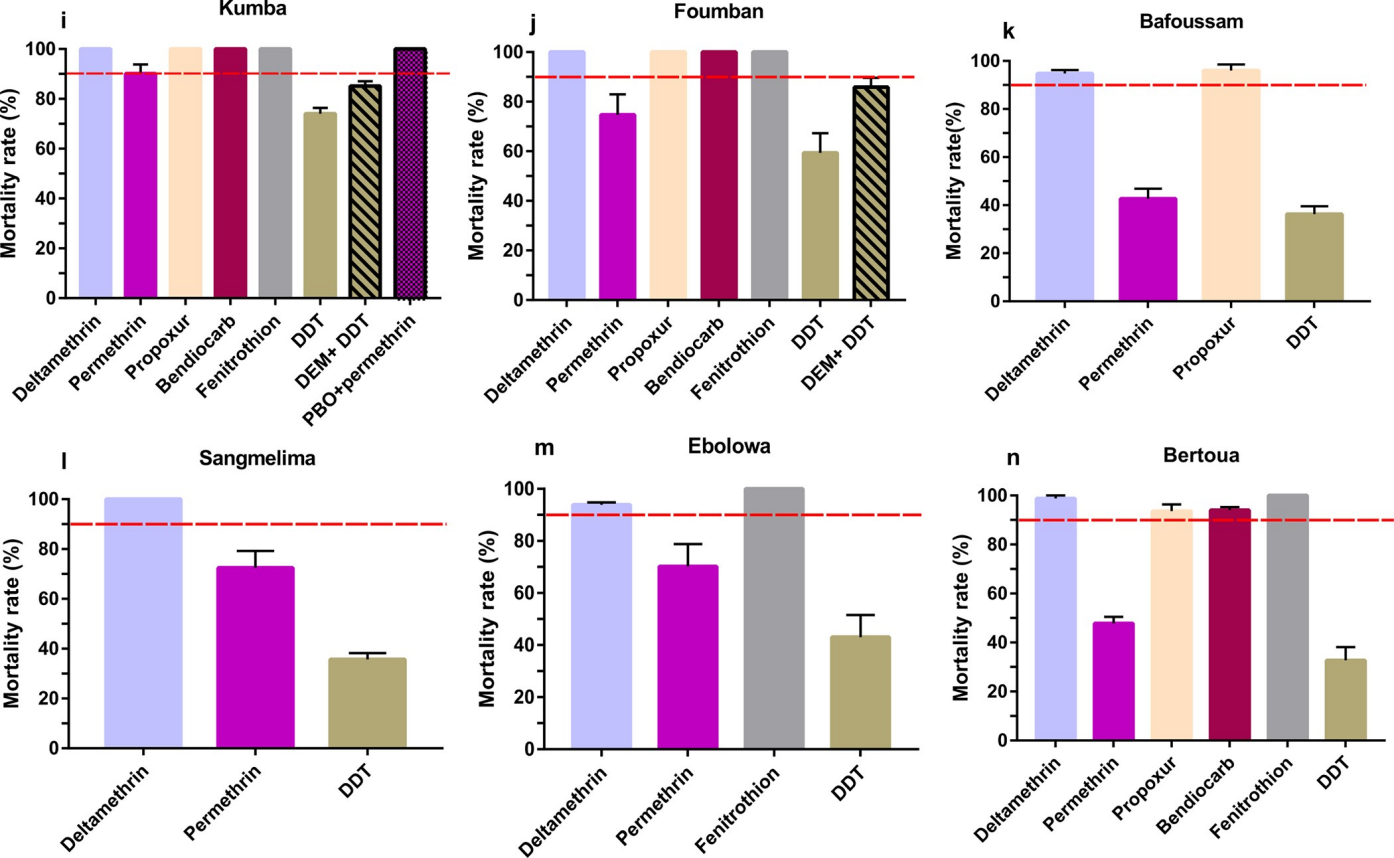
Mortality rates of adult *A*. *albopictus* from Cameroon (6 locations) 24 hrs after exposure to insecticides alone or with 1 h pre-exposure to synergist. i, Kumba; j, Foumban; k, Bafoussam; l, Sangmelima; m, Ebolowa; n, Bertoua. Error bars represent standard error of the mean. DDT, Dichlorodiphenyltrichloroethane. PBO, Piperonyl butoxide. DEM, Diethyl maleate.

**Fig 4:**
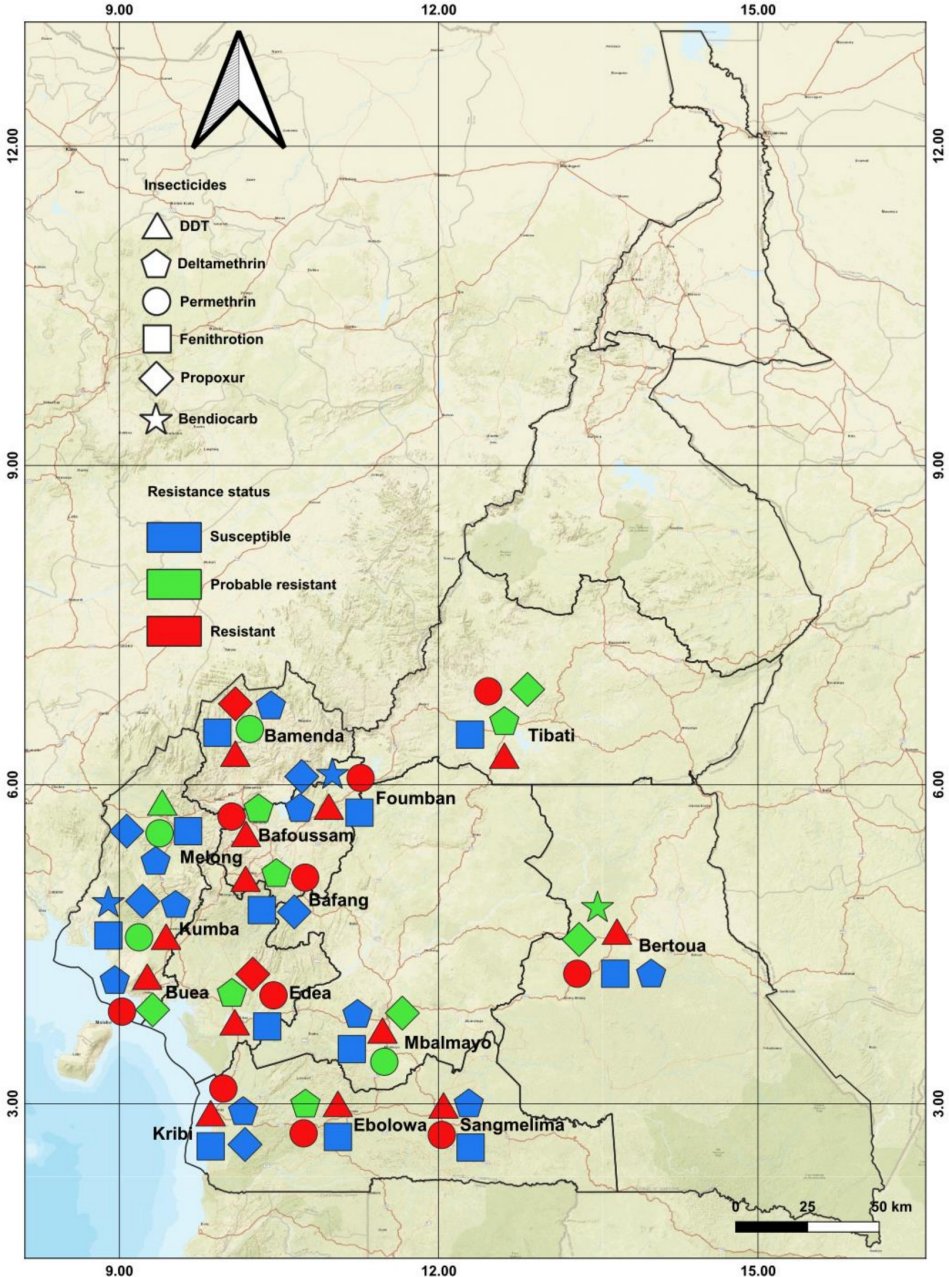
Map of Cameroon showing the insecticide resistance status of *Aedes albopictus* in 14 locations of Cameroon. DDT, Dichlorodiphenyltrichloroethane.

Analysis revealed that 14 populations of *A*. *albopictus* analysed across Cameroon exhibited a high level of resistance to organochlorine DDT with mortality rates ranging from 12.42% in Bafang to 90.03% in Melong (Figs 2 and 3). Resistance was also observed against pyrethroids notably to permethrin (type I) for which nine populations out of 13 were found resistant with mortality rates varying from 34.16% in Bafang to 85.23% in Kribi, probable resistance was found in four populations (Bamenda, Mbalmayo, Kumba, and Melong). In contrast, similar analysis with deltamethrin (type II) revealed that nine populations out of 14 were rather susceptible, and resistance suspected only in five populations with mortality rates ranging from 90.99% in Tibati to 95% in Bafang. Resistance was also reported against carbamates notably to propoxur but only in two populations out of 12 tested, in Edéa and Bamenda, with mortality rates of 66.94% and 88.99%, respectively. However, probable resistance to the carbamate propoxur was found in five populations (Buea, Bafoussam, Mbalmayo, Bertoua, and Tibati) with mortality rates between 94% and 97% while five other populations were susceptible (Figs 2 and 3). In three populations tested with bendiocarb (carbamate), two from Foumban and Kumba were full susceptible and a loss of susceptibility was reported in Bertoua samples. All tested populations across Cameroon exhibited a full susceptibility toward the organophosphate fenitrothion (Fig 4).

**Fig 5:**
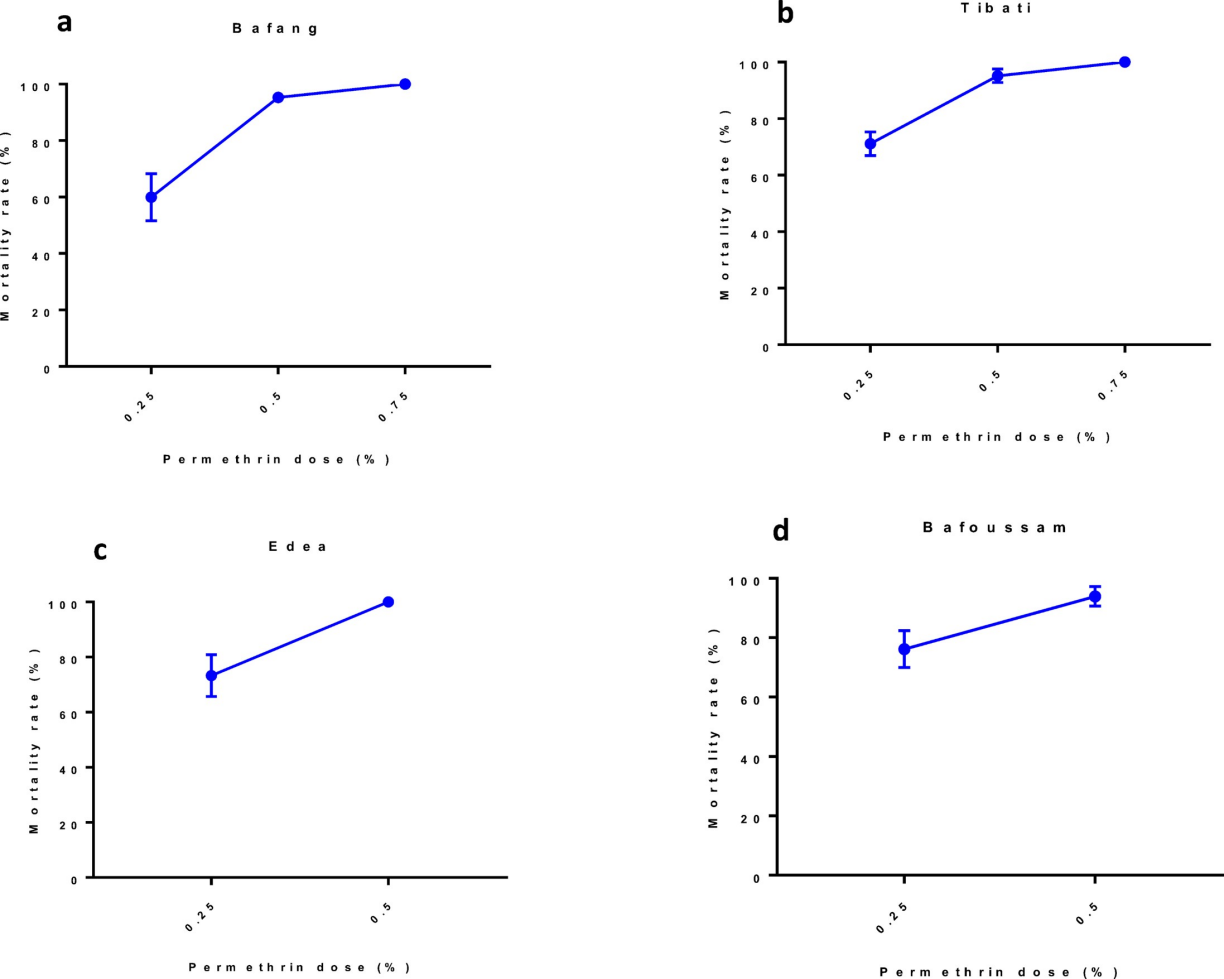
Tests with different doses of permethrin in *A*. *albopictus* from Cameroon. a, Bafang; b, Tibati; c, Edéa; d, Bafoussam.

Using different doses of permethrin (0.25, 0.5 and 0.75%), results showed that the mortality rate increased with dose of permethrin used (Fig 5). Populations of Bafang did not have a full susceptibility even with 2X concentrated permethrin as 100% mortality observed only with 3X. However, population from Edéa exhibit 100% mortality at 2X permethrin.

### Synergist assay

Results from the synergist bioassays showed a full recovery of susceptibility to permethrin after PBO pre-exposure to Kumba population(90.10 ±3.36% mortality without PBO pre-exposure vs 100.0 ± 0.0% mortality after PBO pre-exposure, Chi^2^ *P*> 0.25) while a partial recovery to permethrin was recorded in Buea (76.68± 5.96% mortality without PBO pre-exposure vs 79.76±8.91% mortality after PBO pre-exposure, *P* < 0.75) and Bafang (34.16± 8.93% of mortality without PBO pre-exposure vs 94.58± 2.39% of mortality after PBO pre-exposure, *P* < 0.05) populations, respectively (Figs 2 and 3). The partial recovery of susceptibility to DDT was also reported in Bafang population after pre-exposure to PBO (12.4±4.24% mortality without PBO pre-exposure vs 62.28± 6.59% mortality after PBO pre-exposure, *P* < 0.005). Similar analysis with DEM also induced a partial recovery of susceptibility to DDT with significant difference in Foumban (59.36±7.93% without DEM pre-exposure vs 85.85±7.63% mortality after DEM pre-exposure *P*<0.005), however in Tibati (48.33±12.44% without DEM pre-exposure vs 56.25±2.13% mortality after DEM pre-exposure *P* > 0.25) and Kumba (74.05±2.29% without DEM pre-exposure vs 85.14±1.93% mortality after DEM pre-exposure *P* < 0.25) no significant difference was reported (Figs 2 and 3).

### F1534 *kdr* genotyping

After genotyping of 420 specimens of *A*. *albopictus* from 14 locations across Cameroon no resistant individual was detected. We observed 100% mosquitoes homozygote for the susceptible F1534 allele.

## Discussion

This study investigated the resistance profile of 15 populations of *A*. *albopictus* collected across Cameroon and explored the potential resistance mechanisms involved. Analysis of larval bioassays revealed that all *A*. *albopictus* samples were susceptible to *Bti* and temephos. Similar results were previously obtained in Central Africa notably Cameroon [27], Gabon [27], and Central African Republic [38]. It is important to highlight that data on *Bti* resistance in field populations of mosquitoes are very scarce apart the case of *Culex pipiens* reported in USA [39]. On the other hand, the resistance to temephos has been reported in several countries such as in Greece [40], Malaysia [41,42], and Sri Lanka [43]. Selection of the resistance results from extensive and long-term use of the product incriminated, meanwhile in our knowledge, temephos as well as *Bti* had never been used in vector control program in Central Africa which probably explains the full susceptibility reported for both larvicides in *A*. *albopictus* collected across Cameroon.

Higher level of resistance to DDT was reported in *A*. *albopictus* collected in several places in Cameroon. Indeed, none of the *A*. *albopictus* populations analysed from Cameroon can be considered fully susceptible to DDT, as all populations were either resistant or probable resistant. This result is in accordance with previous data reported in some Central Africa countries especially in Cameroon [28], Central African Republic [38] and the Republic of the Congo [44], and outside Africa: India, Malaysia, and USA [41,45,46]. A significant level of resistance to pyrethroid type I permethrin was reported in nine *A*. *albopictus* populations while for the pyrethroid type II deltamethrin, the resistance was suspected only in five populations. The striking difference of the resistance pattern in both pyrethroids tested could be due to the fact that diagnostic dose used for deltamethrin 0.05% is high than 0.03% recommended for *Aedes* [34] as suggested previously [44]. Indeed, the assessing of intensity of resistance to permethrin using 0.75% permethrin which is the dose recommended for *Anopheles* as 0.05% deltamethrin, populations tested exhibited the full susceptibility. However, the loss of the susceptibility has been previously reported to deltamethrin in Cameroon and Central African Republic [27,28,38]. Although data on permethrin remain scarce in Central Africa preliminary studies in Yaoundé Cameroon highlighted the resistance of this compound [28] using the *Anopheles* diagnostic dose 0.75%. Previous studies in other parts of the world using the diagnostic dose recommended for *Anopheles* had shown that the level of resistance of *A*. *albopictus* to permethrin was higher than those of deltamethrin [41,47]. In contrast, the more recent studies in China highlighted that *A*. *albopictus* is more resistant to deltamethrin than permethrin [43,48]. As in this study, the loss of susceptibility to propoxur and bendiocarb was reported in certain locations across the country [28]. Similar results were observed in Central Africa Republic [38] and outside Africa in *A*. *albopictus* [41,49,50].

The reduced susceptibility to both pyrethroids tested in *A*. *albopictus* may poses a serious threat for vector control programs, because pyrethroids are mainly recommended for the control of adult *Aedes* mosquitoes [51,52]. In addition, it was demonstrated that *A*. *albopictus* was the dominant *Aedes* species in urban area in southern Cameroon [16] and could be considered as main arbovirus vectors in these areas. The source of selection driving the observed resistance to DDT, permethrin, deltamethrin, propoxur and bendiocarb in some *A*. *albopictus* populations remains unclear notably as the use of insecticides against *A*. *albopictus* is limited in the region [27,28,44]. As suggested previously [28,38], domestic used of insecticides through the indoor spraying and impregnating bed nets, and agriculture use could be the main source of resistance selection in *Aedes* vectors in Central Africa. Indeed, the use of pesticides in agriculture for the protection of market gardening could also promoted the emergence of resistance in mosquitoes by contamination of breeding sites and resting places of mosquitoes. As *A*. *albopictus* was firstly reported in Cameroon in early 2000s [14], we cannot exclude the possibility that the invading populations possessed the resistance background, as previously suggested [27,38,44].

A partial or full recovery of susceptibility to permethrin and DDT after pre-exposure to synergist PBO or DEM suggests that the cytochrome P450 monooxygenases and glutathione-S- transferases are playing an important role in the resistance in these populations role in the observed resistance which is consistent with previous data from the sub-region [28,38,44] and in other regions such as in Malaysia where a major P450, *CYP6P12* was associated with pyrethroid resistance [26]. None of the specimens of *A*. *albopictus* genotyped possesses the 1534C allele suggesting this mutation is not currently involved in pyrethroid resistance in populations of this species across Cameroon. Nevertheless, this mutation has been detected in *A*. *albopictus* from several countries outside Africa like Brazil, India, Greece, Singapore, and China [53]. It will be interesting to investigate the role of other *kdr* mutations such as V1016I/G which was described in *A*. *albopictus* in Europe and Asia [54] and investigate the genes involved in metabolic resistance in *A*. *albopictus*.

## Conclusions

Our result showed that the resistance profile varied significantly depending on the insecticides and populations tested. The full susceptibility reported to organophosphates in both larval stages (temephos) and adult stages (fenitrothion) as well as to *Bti* suggests that theses insecticides are suitable for control *A*. *albopictus* across the country. These findings could help to design and implement the best strategies of insecticide-based interventions in Cameroon against arbovirus vector *A*. *albopictus*.
